# Patient perspectives on drug recalls in the Netherlands: a qualitative study

**DOI:** 10.1007/s11096-025-02082-z

**Published:** 2026-01-14

**Authors:** Pieter A. Annema, Lenny M. W. Nahar-van Venrooij, Marcel L. Bouvy, Rob J. van Marum, Hieronymus J. Derijks

**Affiliations:** 1 Department of Pharmacy and Clinical Pharmacology, Jeroen Bosch Hospital, ’s Hertogenbosch, the Netherlands; 2 Department of Elderly Care Medicine, Amsterdam Public Health Research Institute, Amsterdam UMC, Location VUMC, Amsterdam, the Netherlands; 3 Jeroen Bosch Academy Research, Jeroen Bosch Hospital, ’s-Hertogenbosch, the Netherlands; 4 Department of Tranzo, School of Social and Behavioral Sciences, Tilburg University, Tilburg, the Netherlands; 5 Division of Pharmacoepidemiology and Clinical Pharmacology, Utrecht Institute for Pharmaceutical Sciences (UIPS), Faculty of Science, Utrecht University, Utrecht, the Netherlands; 6 Dutch Medicines Evaluation Board, Utrecht, the Netherlands

**Keywords:** Drug recall, Patient perspectives, Patient preferences, Pharmacovigilance, Provider-patient communication, Qualitative research

## Abstract

**Introduction:**

Drug recalls occur regularly with some resulting in medication switches for patients. Limited research into this topic suggests that drug recalls can lead to anxiety and unrest for patients which in turn can affect confidence in and use of medication. In this context, a better understanding of patient perceptions and communication preferences is essential to adequately handle drug recalls and ensure continued medication adherence and trust.

**Aim:**

The aim of this study was to elucidate patients’ experiences, perceptions, and preferences regarding drug recalls.

**Method:**

This qualitative study comprised focus group discussions with patients that experienced a drug recall, recruited through pharmacies from distinct locations in the Netherlands. We aimed to conduct at least two focus groups, each comprising a minimum of six participants. Audio recordings were transcribed verbatim and analyzed using a thematic analysis approach.

**Results:**

It was found that patients had limited knowledge of drug recall procedures, often confused them with shortages, and struggled to interpret associated risks. Communication was frequently perceived as unclear, triggering varied emotional responses and, in some cases, reduced trust in and use of medications. Patients preferred pharmacist-led, personalized communication tailored to recall urgency, and emphasized the importance of shared decision-making, particularly during medication substitutions.

**Conclusion:**

Drug recalls cause a range of emotions in patients, leading to reduced confidence and use of medication in some patients. Preferences centered on understandable, transparent, and personalized communication led by pharmacists. The findings of this study emphasize the importance of embedding patient engagement and tailored communication within pharmacovigilance systems to maintain trust and support shared decision-making during drug recalls.

**Supplementary Information:**

The online version contains supplementary material available at 10.1007/s11096-025-02082-z.

## Impact statements

- The results of this study offer stakeholders valuable insights into how patients experience a recall, how it affects their trust in and use of medication, and their preferences for how recalls are managed and communicated.
- Drug recalls can trigger emotional reactions ranging from reassurance to distress, and in some cases lead to reduced confidence in and use of medications.
- There is a clear need for shared decision-making and personalized drug recall communication co-developed with patients and other stakeholders, ideally delivered by pharmacists.

## Introduction

Drug recalls are an essential instrument employed by drug regulatory agencies to protect patients from unsafe or substandard medicines [1, 2]. A recall is initiated by a marketing authorization holder (MAH), in consultation with national or international authorities, to withdraw a (potentially) defective product from the market. Recalls may result from various defects such as contamination, physical defects, or packaging and labelling problems. They can occur at the distributor, pharmacy, or patient level, depending on risk severity [3].

Patient-level recalls have the greatest impact, as they require notifying patients and usually involve exchanging the affected medication at a pharmacy. In the Netherlands, pharmacists may exchange a recalled drug for the same product form another manufacturer, but substitutions to a different drug or dose require a new prescription. In an earlier study we found that drug recalls led to approximately 855,000 medication exchanges in patients in the Netherlands between 2017 and 2021, including 95,000 substitutions to an alternative drug class [4].

Understanding patients’ perceptions of drug recalls is highly relevant. How patients interpret recall communication, handling, and associated risks may influence their behavior and confidence in medicine quality. Recalls may reassure patients by demonstrating ongoing quality monitoring, but the communication process and medication changes may also undermine confidence, reduce adherence [5, 6], and negatively affect clinical outcomes [7–9].

Despite increasing attention to patient experiences in healthcare, limited research has explored how patients perceive drug recalls and their risks, and existing studies have focused solely on individual drug recalls rather than perceptions of recalls more broadly [10, 11].

### Aim

This qualitative study aimed to address this gap by elucidating patients’ experiences, perceptions, and preferences regarding drug recalls in the Netherlands through focus group discussions.

## Method

### Design and setting

Two focus group discussions were conducted in December 2023 and March 2024 at an outpatient and a community pharmacy, located respectively in a city and a village in different provinces of the Netherlands.

### Design and content of the focus group guide

The guide for the focus group discussions was created by the research group based on a conceptual framework related to medication adherence in patients with hypertension, including social factors, health care system factors, patient factors and disease and treatment factors [12]. Two members from the Jeroen Bosch Hospital (JBZ) digital panel who had experienced a recall piloted the initial version, and their feedback confirmed that the discussion topics were relevant and that no important topics were missing. The JBZ digital panel includes about 2000 patients, relatives, or caregivers participating anonymously in digital questionnaires. Prior to the second focus group, the guide was revised to include the topic risk perception. A translated version of the final guide is provided in Appendix I.

Each session began with an explanation of the procedure, a definition of drug recalls, and participant introductions. Participants then discussed their experiences with drug recalls and their views on the associated communication. Subsequent topics explored the recall’s impact on medication adherence, confidence in medicines, and trust in healthcare providers, as well as patients’ preferences regarding communication and management approaches. Sessions concluded with participants highlighting aspects they deemed most significant.

### Participants and recruitment

Patients aged ≥ 18 years who had personally experienced a drug recall were eligible to participate. Distinct recruitment methodologies were employed for each focus group discussion. For the initial meeting, posters and flyers were displayed in the outpatient pharmacy and community pharmacies surrounding the city of ‘s-Hertogenbosch. Patients could contact the researcher through phone or email or by notifying their community pharmacy. This recruitment effort yielded no participants; therefore, a purposive sampling strategy was implemented. The goal was to invite participants of varying ages and genders who had experienced different drug recalls. Eligible patients were identified from JBZ outpatient pharmacy records, which detailed patients contacted about a drug recall. In addition, the two JBZ digital panel members who were previously involved in the pilot expressed their interest in participating. One of the researchers (PA) contacted all eligible participants to provide study information and confirm eligibility.

For the other focus group, purposive sampling was combined immediately with posters and flyers. Potential participants were identified from community pharmacy records and approached by a researcher (PA) for study information and eligibility screening until a sufficient number agreed to participate. All participants received a participant information sheet and provided written informed consent before participation. No compensation was offered.

### Data collection and processing

The focus groups lasted 2–2.5 h and were attended by participants, moderators (JD, PA) and, during the second focus group meeting, additional researchers (MB, RvM). One researcher (PA) had prior contact with participants during recruitment, while other researchers had no prior contact with participants. Sessions were audio-recorded and transcribed verbatim by a professional transcription service, after which a researcher (PA) checked the transcripts for accuracy. Identifying information was removed to ensure anonymity.

### Data analysis

Data were analyzed using a thematic analysis approach using ATLAS.ti v24. Field notes were used to support the transcripts. Researchers (PA, LNV, and JD) thoroughly reviewed both transcripts to familiarize themselves with the content. The initial quarter of the transcript from the first focus group discussion was independently open-coded by the three researchers, followed by discussions to resolve disagreements and reach consensus. One researcher (PA) completed coding both transcripts, consulting regularly with two others (LNV and JD). Codes were subsequently organized into themes and subthemes by PA and reviewed by LNV and JD, incorporating topics from the focus group guide where relevant. Findings were presented and discussed with the full research team (PA, LNV, MB, RvM, and JD) to enhance reliability and to assess whether saturation was reached. Several studies indicate that data saturation in qualitative research using thematic analysis can be achieved with 12 participants [13, 14]. We therefore aimed to perform at least two focus groups, each comprising a minimum of six participants. Quotes deemed relevant for inclusion in this manuscript were translated from Dutch to English by PA and reviewed by the other researchers. The Standards for Reporting Qualitative Research (SRQR) was used to draft this manuscript [15].

### Researcher characteristics

PA (♂), a PhD candidate and hospital pharmacy resident, completed two qualitative research courses; LNV(♀), holding a PhD, is a clinical epidemiologist with experience in qualitative research; MB (♂),professor and pharmacist, is a member of the Medicines Evaluation Board (MEB) with qualitative research experience; RvM (♂), professor and geriatrician, is a former member of the MEB with qualitative research experience; JD(♂), professor and hospital pharmacist, completed a qualitative research course.

### Ethics approval

The Medical Ethics Review Board METC Brabant (reference ID: NW2023-69) waived ethical approval as the study fell outside the scope of the Dutch Medical Research Involving Human Subjects Act (WMO).

## Results

### Participants

Thirteen patients (ages 53–73) participated in two focus group sessions, including five women and eight men (Tab. 1). Educational backgrounds (classified using the International Standard Classification of Education (ISCED) [16]) varied, though the majority had completed higher education. On average, participants used five different medicines. During the introductory rounds, participants expressed varying basic attitudes toward healthcare, ranging from highly positive to negative. Several participants said they joined this study to give back as an appreciation for care they had received.

**Table 1:** Participant characteristics

	Focus group 1	Focus group 2
Setting	Outpatient pharmacy in a city	Community pharmacy in a village
Number of participants *(n)*	7	6
Female participants *(n)*	2	3
Age of participants (median, range), years	68 (63–72)	65 (53–73)
*Level of education*^**a**^		
Low (0–2)	–	2
Medium (3–4)	4	–
High (5–8)	3	4
*Medicines in use*		
0–5	2	3
6–10	2	3
> 10	2	–
Not disclosed	1	–
*Type of medication*		
Direct effect on disease	1	–
Prevention of disease	–	3
Both	6	2
Not applicable	–	1
*Recalled medication*^b^		
ARB	4	2
Epinephrine auto-injector	1	1
Ranitidine	1	3

*ARB* Angiotensin Receptor Blocker

^a^Level of education is classified based on the International Standard Classification of Education (ISCED)

^b^One participant (R4, FG1) learned during the focus group discussion that he had not, in fact, been affected by a recall

Participants shared experiences with recalls involving histamine H2-receptor antagonists, antihypertensives, and epinephrine auto-injectors. These drugs were recalled due to various drug defects, including the presence of carcinogenic impurities and device malfunctions. One participant (R4, FG1) learned during the session that he had not actually been affected by a recall, and therefore did not meet the eligibility criteria. After group discussion, the patient continued participation while reflecting hypothetically.

### Themes and subthemes

**Fig. 1:**
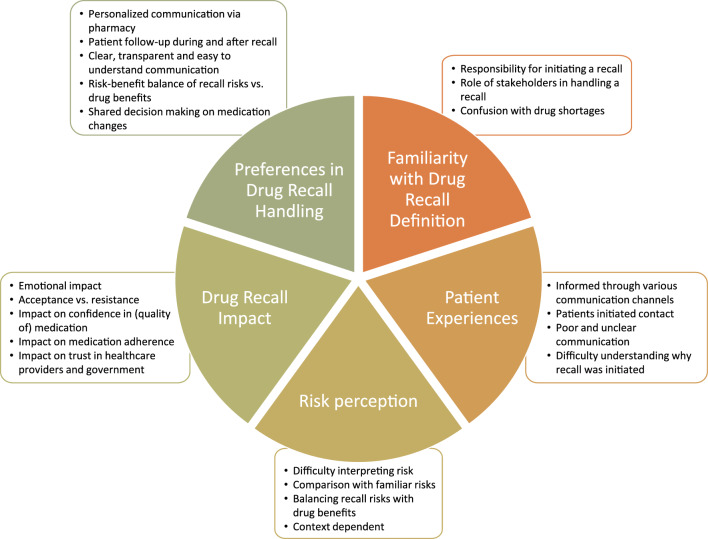
Overview of the themes and their subthemes derived from the focus group discussions

Thematic analysis yielded five main themes: Familiarity with Drug Recall Definition, Patient Experiences, Risk Perception, Drug Recall Impact, and Preferences in Drug Recall Handling, each with corresponding subthemes (Fig. 1). The following sections address each (sub)theme, supported by relevant quotes.

### Familiarity with definition of drug recall

Participants were generally unfamiliar with recall procedures and unaware of *who is responsible* for initiating a recall, and *the roles of governmental bodies, manufacturers, and healthcare professionals*. Some participants *confused drug recalls* with medication changes due to drug shortages, even after clarification from the moderator.

‘I agree with the lady. That you can also consider that [a change in medication due to a drug shortage] as a recall.’ (R1, FG1)

Most participants had experienced non-recall-related medication switches, which many viewed negatively due to side effects or reduced effectiveness.

### Patient experiences

*Notification methods varied:* some participants were contacted directly by their pharmacy via letter, email, or telephone, while others first heard through media (television, internet or newspapers). The latter often *initiated contact* with their healthcare providers for guidance. Pharmacies asked patients to return their medication for batch verification and provided a new batch or therapeutic alternative if needed.

Many participants found the communication *vague or incomplete*, causing uncertainty about urgency and required actions.

‘And if I could come along with the medication, because then they could see from the lot number, again, I don't know what lot means, but they could see from the number whether it was from that batch.’ (R6, FG1)

Participants often *did not understand the reason for the recall*, in particular in cases involving drug contamination. Whether the contamination referred to the drug, the packaging, or some other aspect of the product. As a result, several participants referred to and sought clarification regarding the recall’s significance and associated health risks from healthcare-professional acquaintances.

### Risk perception

Participants often *struggled to interpret recall-related risks*. Several felt uncapable to interpret the implications of an impurity themselves and entirely relied on healthcare professionals. Others *compared the issue with familiar risks*, such as lifestyle or environmental exposures, but these analogies (also used by Dutch authorities) did not resonate with all participants.

‘Yeah, well, like you would eat burnt meat or something. I said: well I don't eat that […] I don't barbecue anyway. But uh, I don't like that [comparison].’ (R2, FG2)

Other participants *weighed the theoretical risk of harm* against the perceived therapeutic necessity of the drug.

‘But you are less concerned about the risk of cancer, I understand, because it [the medication] is so important to you to, well, prevent that stroke?’ – ‘Yes, to not end up in a wheelchair… Exactly.’ (R2, FG2)

Risk perception was found to be *context-dependent*. Participants' views varied based on multiple factors, including medication purpose (e.g., chronic vs. as-needed use), availability of alternatives, and nature of the defect. The perceived importance and therapeutic role of the drug shaped how the risk was tolerated.

‘Isn't it sometimes also a risk to recall everything? I mean: what if a medicine is no longer available? I know that there was a period when there were too few EpiPen’s. Then I think: then you die sooner because you no longer have an EpiPen, then if you use a pen that is perhaps a bit contaminated, or that you need a few more because it doesn't work properly. And that is probably the case with a lot of medicines.’ (R1, FG2)

### Drug recall impact

The impact of drug recalls varied among participants. Some reported minimal disruption; others became more vigilant, researching their medication use or keeping recall letters as a safeguard in case future issues emerged.

The *emotional impact* was substantial for several participants: Learning about possible carcinogenic impurity in their medication caused considerable distress, fear, and uncertainty. Moreover, participants emphasized that the anxiety may have been disproportionate to the actual risk, yet no less impactful.

‘Because I think that a lot of people are scared to death, while there is relatively little going on. And that is of course also super annoying. Because if you get really scared of something, it can perhaps have a bigger impact than if you keep taking that medicine.’ (R4, FG2)

Despite these concerns, some participants were satisfied with the recall handling. They appreciated direct contact from the pharmacist and felt reassured that the issue had been detected, even after many tablets had already been consumed.

‘Yes, I thought it was good that they called me. […] Yes, then you know it right away. Because imagine that you get a letter, then I think: yes, how long is that letter on the way? Why… why don't they tell you right away?’ (R2, FG2)

*Acceptance of proposed medication changes differed.* Some viewed the recall as an isolated incident, others were reluctant to accept changes to their medication. One participant preferred to continue using the recalled product due to its effectiveness, while others questioned the pharmacist’s advice or insisted that their physician be involved in therapeutic decisions.

The impact on patient *confidence in medication* in general was heterogeneous. For some, the recall had little to no effect, while for others, it led to diminished or even complete loss of trust in medicines. The degree of impact appeared to depend on the specific cause of the recall and whether the recall was an isolated incident or would occur repeatedly with the same manufacturer.

‘And then you don't trust it all that much anymore. So, for me that recall was, yes, not nice. And I also became more suspicious of other medicines. Like: if that is contaminated, that can be too. […] It didn't increase trust. It rather fueled suspicion.’ (R6, FG1)

Most respondents indicated that the recall did not alter their *medication use*, but became more vigilant and critical of their medicines. For some, the drug’s perceived necessity, outweighed recall-related concerns, supporting continued adherence. In contrast, one participant stopped taking the medication due to concerns about a minor cancer risk, despite enduring persistent pain.

‘Well, I finally said and then I was in a lot of pain myself, you know. But I didn't go back to the GP for replacement tablets. Because I thought: well, I'm just not going to do that.’ (R5, FG2)

Overall, participants reported a strong baseline level of *trust* in their pharmacists, GPs, and medical specialists. Most stated that the drug recall had not affected their relationship with these professionals, as they did not blame them. One participant, however, reported reduced trust due to poor communication.

‘I'm still really angry about that. And also, about the lack of communication. I just wanted openness, like: tell me what's going on, then I'll understand. But make no mistake, I'm not a stickler. So, it definitely had an impact on my relationship with the pharmacist.’ (R6, FG1)

Participants views regarding the impact on *trust in governmental authorities* varied. Some appreciated the oversight of drug quality and felt that recalls reinforced their trust in regulatory systems. Others reported no change or continued distrust, citing concerns about influence from the pharmaceutical industry and broader political dissatisfaction extending beyond healthcare.

### Patients’ preferences regarding future recall communication and handling

Participants consistently highlighted *personalized communication* during drug recalls as most important, identifying pharmacists as the most important and trusted source. For large-scale recalls or those with widespread impact they also valued media involvement to ensure broad public awareness. Several participants found it difficult to interpret health implications and highlighted the need for additional resources such as helplines or dedicated websites.

Preferred communication modes (telephone, email, letter) depended on the importance of medication and urgency of the recall. Life-saving drugs, such as epinephrine auto-injectors, warranted rapid and direct contact, while recalls of preventive medication could be communicated through slower channels. Moreover, for recalls involving potentially serious consequences, such as a cancer risk, participants found impersonal communication unacceptable and stressed the need for in-person explanation.

‘If I am indeed prescribed something by a professional, and that professional has, so to speak, recommended it to me, and I take it, and it turns out to be contaminated, with a possible risk of cancer, then I want it to be explained to me properly, what that risk is. And not by receiving a letter on my doormat.’ (R5, FG2)

Participants furthermore stressed the need for *follow-up* to ensure that all patients received and understood recall information. They viewed letters and emails as unreliable for some groups, particularly older adults or those less engaged with mail or digital communication.

‘[…] Well, I have a mother-in-law, who is a few years older than me. But she doesn't empty the mailbox every day, right? […] And she doesn't read emails either. So, she just gets that information… yes, coincidentally, when I ask her that, right? […] So, I don't really think sending a letter is [laughs] entirely sufficient.’ (R2, FG1)

Beyond immediate recall actions, participants wanted updates following the initial recall process on whether recalled medication would eventually become safe to use again.

Regarding content, participants emphasized *clarity, transparency, and contextualization*. Communications should describe the nature of the defect, associated risks, and how *risks compared with the therapeutic benefits* of the drug in a manner that supports informed decision-making. When substitution was necessary, they wanted explicit information on whether the alternative was pharmaceutically equivalent.

‘And of course, in simple language, so that I understand it, I am not a pharmacist. So just be clear.’ (R6, FG1)

The degree to which participants wished to engage in *shared decision making* varied. Some deferred entirely to healthcare professionals, whereas others wanted to be actively involved and fully informed, enabling them to make well-informed decisions about discontinuing the affected medication or accepting an alternative.

‘Yes, definitely. I think it's just really easy when you know what it's about, that you can make that choice yourself. But then the trust is also greater, because then your pharmacist has explained why. And then it's also a bit easier to talk. And then it’s also easier to make a choice.’ (R1, FG1)

Participants expected that when a change in medication was necessary, particularly involving a different product rather than a different batch, the prescribing physician would be consulted or at least notified.

When asked whether pharmacists should not bother patients with a recall and just silently replace affected stock, responses were mixed. Some felt explicit communication was unnecessary when risks were minimal.

‘Yeah, but what does it matter if you happen to take those 30 more pills, and you get the right one next time? Because that… that's what it's all about.’ (R6, FG2)

Others strongly preferred transparency and would feel misled if not informed.

‘I would feel cheated if you guys knew that and then didn't….’ (R5, FG2)

## Discussion

This qualitative study explored patients' experiences, perceptions and preferences regarding drug recalls in the Netherlands. Participants showed limited baseline knowledge of recall procedures, often confusing recalls with issues such as drug shortages, and many struggled to interpret recall-related risks. Communication—particularly regarding its urgency, rationale, and health implications—was often perceived as unclear. Emotional responses ranged from reassurance to distress, with some participants reporting reduced trust in medications, healthcare providers, or regulators. Participants became more vigilant in their medication use, and one discontinued treatment due to perceived risk. Participants expressed a strong preference for personalized, transparent, and context-sensitive communication, ideally initiated by pharmacists and tailored to the urgency of the recall, as well as for shared decision-making especially when substitutions were required.

Drug recalls elicited anxiety and uncertainty, a response consistent with previous research [10]. Participants described becoming more vigilant and critical toward their medicines, reflecting similar patterns noted by others [10, 11]. Recall communication was found unclear, which is consistent with evidence that patient information leaflets often lack sufficient clarity [17]. However, unlike Hawker et al., who found the media to be the preferred source of information, participants in our study favored communication from pharmacists. This contrast may be attributable to the nature of the recall examined by Hawker et al., in which rofecoxib was suddenly and completely withdrawn from the market, resulting in extensive radio and news coverage before pharmacists had the opportunity to communicate with patients. Participants in our study preferred that recalls of this magnitude should first be communicated by the pharmacy and subsequently by the media.

Communication was viewed as the most important factor, with risk communication playing a central role. While strategies such as contextualizing risks and presenting absolute risks were used in practice, participants indicated that other recommended approaches, such as visual aids, discussion of baseline risks, and checking patient understanding, were not applied [18, 19]. Given that most participants struggled to interpret risks, these gaps represent opportunities to enhance risk comprehension and strengthen shared decision-making by tailoring risk communication to patients’ levels of education and age, and by implementing additional recommended strategies [18–21].

Previous research has applied the Health Belief Model (HBM) to understand patient responses to recalls, conceptualizing recalls as health threats that require individual action [22, 23]. Several HBM constructs were reflected in our findings, although our analysis was not limited to the HBM and also considered additional contextual and behavioral factors. *Cues to action* were evident in how patients were notified, shaping perceived urgency and subsequent steps. *Perceived barriers* included concerns about switching medications, such as uncertainty regarding the availability and equivalence of alternatives. *Perceived severity* was generally acknowledged, while *perceived susceptibility* varied, with some patients viewing a risk of 1 in 100,000 as meaningful and others considering it negligible. *Perceived benefits* also differed: some patients felt replacement was clearly advantageous, whereas others were uncertain due to low perceived susceptibility or reluctance to incur additional pharmacy costs. Moreover, some participants wished to continue using recalled medication despite known risks, citing perceived therapeutic benefits, an observation consistent from previous studies [11]. *Perceived self-efficacy* was less apparent, as patients have limited ability to address issues such as defective devices or contaminated medications themselves.

### Strengths and limitations

This study offers valuable insights into patients’ experiences, perspectives and preferences regarding drug recalls. The inclusion of participants from two distinct regions of the Netherlands, with varied educational backgrounds, exposed to different drug recalls enhances the robustness and credibility of the findings. Our results draw attention to the substantial emotional impact of drug recalls, variation in risk interpretation, and the need for personalized, transparent communication strategies. The findings contribute to the growing recognition that patient engagement is essential in pharmacovigilance [24].

Several limitations must be considered. While the qualitative design enabled a detailed exploration of patient perspectives, the sample was small and largely homogeneous, consisting mainly of older Dutch native citizens, which may limit transferability. Nonetheless, this demographic is highly relevant, given older individuals have the highest medication use and greater likelihood of being affected by recalls [25]. Moreover, the small sample proved sufficient as no new themes emerged during the second session. One participant did not meet eligibility criteria, but their presence did not appear to influence findings and their quotes were not used in this manuscript.

Despite initial efforts to recruit participants from varied cultural backgrounds, anticipating potential variations in perspectives due to differences in health literacy, inclusion was limited. However, both expert consultation and prior research indicate that health literacy disparities are more strongly associated with education, language proficiency, and communication barriers rather than ethnicity per se [26–29].

Conducting the study in the Netherlands, where health literacy is relatively high, may further limit generalizability. In lower-health literacy settings, difficulties understanding drug recall information may be more widespread [30]. Finally, recall bias is a possible, as participants reflected on past experiences of varying recency, which may have introduced inconsistencies.

### Recommendations

The findings underscore the need for patient-centered strategies for communicating and managing drug recalls. Communication should be accessible, use plain language, and delivered through various formats (e.g. text, video, visuals). Personal communication is warranted for recalls involving life-sustaining treatments, perceived serious health risks (e.g., carcinogenicity), or medication substitutions that extend beyond brand changes. While most participants reported that their trust in healthcare providers remained intact, trust in governmental institutions was more negatively affected. Although no simple solution exists, opportunities lie in clear communication and patient involvement. Pharmacists are well positioned to deliver such tailored communication, facilitate shared decision-making, and coordinate with prescribers.

Regulators, manufacturers, and professional associations should adopt a coordinated, multi-stakeholder approach to recall management, embedding patient perspectives in the design of recall interventions and communication. In practical terms, by engaging patients in creating drug recall communication materials, pharmacies can be equipped with communication tools that better meet patients’ needs. Enhancing shared decision-making, especially when medication substitutions are proposed, may improve medication adherence and reduce emotional distress.

Future research should assess the generalizability of these findings, ideally through quantitative methods. Additional studies are also warranted to better understand patients' risk perceptions and to determine what level of risk is deemed acceptable in relation to therapeutic benefits during recall situations.

## Conclusion

This qualitative study provides new insights into how patients perceive and respond to drug recalls, highlighting the importance of patient engagement in pharmacovigilance. Patients showed limited knowledge of recall procedures, difficulties interpreting risks, and often experienced recall communication as unclear, leading to varied emotional responses and, in some cases, reduced trust in and use of medications. Patients expressed a strong preference for personalized, transparent, and pharmacist-led communication, as well as shared decision-making regarding therapy changes. These findings emphasize the need to integrate patient engagement into pharmacovigilance systems, with tailored communication strategies that support effective recalls while aligning with patient-centered care principles.

## Supplementary Information

Below is the link to the electronic supplementary material.

Supplementary file1 (DOCX 28 kb)

## Data Availability

This study analyzes qualitative data and the participants did not consent to have their full transcripts made publicly available. Supporting excerpts from the raw data (quotes from focus groups with participants) are available within the text of the paper. Due to ethical restrictions protecting participant confidentiality, the full transcripts of focus groups will not be publicly available. Instead, anonymized excerpts of the transcripts will be available upon reasonable request from the corresponding author.
