# Supplementary material for: Patient perspectives on drug recalls in the Netherlands: a qualitative study
Source: Int J Clin Pharm. 2026 Jan 14;48(3):864–73. doi: 10.1007/s11096-025-02082-z (PMC13176037; doi:10.1007/s11096-025-02082-z)
Supplement: Supplementary file 1 — Supplementary file1 (DOCX 28 kb) [file 11096_2025_2082_MOESM1_ESM.docx]

**Patient Perspectives on Drug Recalls in the Netherlands: a Qualitative Study**

**Journal:** International Journal of Clinical Pharmacy

**Authors:** Pieter A. Annema^1,2^, Lenny M. W. Nahar-van Venrooij^3,4^, Marcel L. Bouvy^5,6^, Rob J. van Marum^1,2^, Hieronymus J. Derijks^1,4^

1. Department of Pharmacy and Clinical Pharmacology, Jeroen Bosch Hospital, ‘s-Hertogenbosch, the Netherlands
2. Department of Elderly Care Medicine, Amsterdam Public Health Research Institute, Amsterdam UMC, Location VUmc, Amsterdam, the Netherlands
3. Jeroen Bosch Academy Research, Jeroen Bosch Hospital, ’s-Hertogenbosch, the Netherlands
4. Department of Tranzo, Tilburg University, School of Social and Behavioral Sciences, Tilburg, the Netherlands
5. Division of Pharmacoepidemiology and Clinical Pharmacology, Utrecht Institute for Pharmaceutical Sciences (UIPS), Faculty of Science, Utrecht University, Utrecht, the Netherlands
6. Dutch Medicines Evaluation Board, Utrecht, the Netherlands

**Corresponding author:** Pieter A. Annema, e-mail: p.annema@jbz.nl

**Appendix I: Topic guide**

Welcome and introduction of the focus group

- Moderator welcomes participants to the focus group discussion
- Researchers are introduced including their role during the focus group discussion
- Moderator explains the goal of the focus group discussion
  - Today’s meeting is about drug recalls. You’re probably aware that in recent years there have been issues with the availability of medicines. One of the reasons for this is drug recalls. A drug recall happens for example when the supplier discovers that the quality of a drug does not meet the standards they have set for it. The medicines that have already been delivered to patients are then taken back. You may also be familiar with this from other types of products. For example, supermarkets sometimes recall a product. This doesn’t necessarily mean the product is dangerous, but they prefer not to take any risks.

Today, we’d like to use this group discussion to learn about patients’ experiences with drug recalls and how such a recall affects you. Additionally, we want to gain insight into your preferences regarding communication around a recall. The results of this meeting, along with the results from other sessions, will be compiled and published in a scientific article.

- Moderator explains the procedure of the focus group discussion
  - The discussion, including a short break, will last a maximum of 2 to 2.5 hours. We have prepared a number of questions related to medicine recalls. The goal is for you to share your ideas, opinions, views, and experiences with each other and with us. You don’t need to agree with one another as we’re interested in all perspectives, but it is important that everyone treats each other with respect and lets others finish speaking. The intention is to encourage as much discussion among the group as possible; the moderator is there only to keep the conversation moving, ask questions, and occasionally guide the discussion. There are no right or wrong answers.
- To process the content of the discussion as accurately as possible afterward, we will record the conversation. These recordings will be treated confidentially.
- Everything that is said will be processed confidentially and anonymously (coded). Your pharmacist, GP, or other healthcare providers will not know what you have said here.
- The moderator asks participants to briefly introduce themselves (name, age, background – education/work, and motivation for participating in this meeting)

Opening question

What is your personal experience with a medicine recall, and how did you experience that recall?

- Specifically regarding communication:
  - Did you feel you were informed in time?
  - Did you receive information about what the issue was?
  - Did you receive information about any possible risks?
  - Did you receive information about an alternative medicine?

Trust

- How does a medicine recall affect your trust in medicines?
  - The specific medicine that was recalled
  - Medicines in general
- Do you change the way you use your medicines because of a recall?
  - The specific medicine that was recalled
  - Medicines in general
- How does a drug recall affect your relationship with healthcare providers: pharmacy/GP/specialist?
- How does a drug recall affect your trust in the government?

Risk perception

Imagine you receive a message stating that a medicine you are using contains a substance that exceeds the allowed limit. As a result, the risk of developing cancer may be increased. For example: if 100,000 patients were to use the maximum dose of this medicine for their entire life, one additional person would develop some form of cancer.

- How does this message affect you? Please explain.
- What would you do in response? Please explain.
  - Continue using the medicine
  - Switch to another brand with the same active ingredient
  - Use a completely different medicine
  - Another option?
- Does the nature or severity of the condition you are treating with this medicine affect your decision?
- Would your decision be different if you knew that the medicine prevents a heart attack in 1 out of 100 people?

Patient preferences

- When would you want to be informed about a recall?
  - Patient level vs. pharmacy/wholesaler level
  - How detailed should the information be? Does this depend on the level of the recall?
- What do you need in order to decide whether or not to continue using a medication after a recall?
  - What kind of information do you need?
  - Do you seek advice or support from your social environment? If so, from whom?
  - When do you feel confident enough to make a decision?
  - Who would you prefer to inform you about a recall?
    - Pharmacy / General Practitioner / Government / Media

Conclusion of the discussion

- Write down one thing you consider most important regarding a drug recall.
- Briefly explain why this is most important to you.
- Are there any remaining questions?
